# Pharmacological Cognitive Enhancement in Healthy Individuals: A Compensation for Cognitive Deficits or a Question of Personality?

**DOI:** 10.1371/journal.pone.0129805

**Published:** 2015-06-24

**Authors:** Larissa J. Maier, Michael D. Wunderli, Matthias Vonmoos, Andreas T. Römmelt, Markus R. Baumgartner, Erich Seifritz, Michael P. Schaub, Boris B. Quednow

**Affiliations:** 1 Swiss Research Institute for Public Health and Addiction (ISGF), Associated Institute at the University of Zurich and WHO Collaborating Centre, Zurich, Switzerland; 2 Experimental and Clinical Pharmacopsychology, Department of Psychiatry, Psychotherapy and Psychosomatics, Psychiatric Hospital of the University of Zurich, Zurich, Switzerland; 3 Center of Forensic Hairanalytics, Institute of Forensic Medicine, University of Zurich, Zurich, Switzerland; 4 Department of Psychiatry, Psychotherapy, and Psychosomatics, Psychiatric Hospital University of Zurich, Zurich, Switzerland; 5 Neuroscience Center Zurich, University of Zurich and Swiss Federal Institute of Technology, Zurich, Switzerland; University G. D'Annunzio, ITALY

## Abstract

The ongoing bioethical debate on pharmacological cognitive enhancement (PCE) in healthy individuals is often legitimated by the assumption that PCE will widely spread and become desirable for the general public in the near future. This assumption was questioned as PCE is not equally save and effective in everyone. Additionally, it was supposed that the willingness to use PCE is strongly personality-dependent likely preventing a broad PCE epidemic. Thus, we investigated whether the cognitive performance and personality of healthy individuals with regular nonmedical methylphenidate (MPH) use for PCE differ from stimulant-naïve controls. Twenty-five healthy individuals using MPH for PCE were compared with 39 age-, sex-, and education-matched healthy controls regarding cognitive performance and personality assessed by a comprehensive neuropsychological test battery including social cognition, prosocial behavior, decision-making, impulsivity, and personality questionnaires. Substance use was assessed through self-report in an interview and quantitative hair and urine analyses. Recently abstinent PCE users showed no cognitive impairment but superior strategic thinking and decision-making. Furthermore, PCE users displayed higher levels of trait impulsivity, novelty seeking, and Machiavellianism combined with lower levels of social reward dependence and cognitive empathy. Finally, PCE users reported a smaller social network and exhibited less prosocial behavior in social interaction tasks. In conclusion, the assumption that PCE use will soon become epidemic is not supported by the present findings as PCE users showed a highly specific personality profile that shares a number of features with illegal stimulant users. Lastly, regular MPH use for PCE is not necessarily associated with cognitive deficits.

## Introduction

Prescription stimulants such as methylphenidate (MPH) are controversially discussed as potential drugs for pharmacological cognitive enhancement (PCE) in healthy individuals [1–5]. The increase of MPH prescriptions in the past two decades was supposed to coincide with an increased nonmedical use of MPH for PCE and relatively high prevalence rates of PCE among college students seemed to confirm the prediction [6,7]. However, many studies failed to clearly define “nonmedical use” and considered different substances for PCE impeding the interpretation of the results [8]. Nevertheless, PCE is much more prevalent in the United States compared to Europe [8], but in both regions, MPH is the most frequently misused prescription drug for PCE [1,9].

MPH elevates the neurotransmission of dopamine and noradrenalin by reuptake inhibition at the respective monoamine transporters, and was proposed to influence executive functions and working memory in healthy individuals [10,11]. However, potential benefits and risks of PCE are both modulated by individual differences in response to drugs further depending on drug dose and task requirements. Consequently, procognitive effects of MPH are baseline-dependent (e.g., amelioration at low and impairment at high baseline performance) and afflicted with several trade-offs (e.g., improvement in one cognitive domain with the cost of impairments in other cognitive domains) as well as psychiatric side-effects [10–14].

The use of MPH for the treatment of ADHD is well-established and the potential side-effects are justified by the proven effectiveness [15]. However, this does not reclaim the use by healthy individuals without cognitive deficits. So far, it is unclear whether regular MPH use for PCE in healthy individuals is related to negative long-term cognitive, psychopathological, and neurobiological consequences [10,14]. Nonetheless, previous studies found a higher prevalence of PCE among students with lower grades [1,16,17]. In general, the misuse of prescription stimulants might be associated with neuropsychological deficits prior to or as a consequence of PCE. Reske and colleagues found that occasional prescription stimulant users showed enhanced verbal fluency but, at the same time, more deficits in verbal learning, memory, and cognitive flexibility compared to stimulant-naïve controls. Therefore, they suggested that pre-existing cognitive deficits and subtle executive dysfunctions might be predictors for stimulant use [18,19]. On the other hand, PCE itself might cause drug induced cognitive impairments as shown in a recent longitudinal study with recreational cocaine users [20]. Accordingly, the use of MPH might affect neuroplasticity and may, therefore, alter cognitive function, behavior, and personality of users [21].

Like amphetamine, MPH is a phenylethylamine derivate but shares the mechanism of catecholamine reuptake inhibition with cocaine [10]. The Zurich Cocaine Cognition Study (ZuCo2St) revealed that not only dependent but also recreational cocaine users showed significant deficits in the cognitive domains of attention, working and long-term memory, and executive functions [22]. Cocaine users also revealed higher levels of self-reported impulsivity and novelty seeking and more ADHD symptoms compared to stimulant-naïve controls [22,23]. Moreover, cocaine use was associated with reduced neural sensitivity to social reward potentially explaining the users’ deficits in social interactions such as less emotional empathy and a smaller social network [24,25]. Studies considering the nonmedical use of MPH in healthy individuals have only addressed acute MPH effects on social cognition and behavior [26,27], while the effects of chronic MPH use on social behavior are unknown so far.

The bioethical debate on neuroenhancement is based on the assumption that the use of putatively neuroenhancing stimulants already appears to be highly popular and that PCE use will further spread in the near future. However, these assumptions have been recently disputed [14,28]. One argument against a future epidemic of PCE is that not everyone is equally interested in cognitive enhancement [29] assuming that personality has an essential impact on the willingness to use PCE [14]. Surprisingly, the influence of personality on the preference of PCE has scarcely been investigated yet, but Quednow proposed that in particular narcissistic and ambitious people might be more interested in PCE [14]. Preliminary data recently suggested that PCE is positively associated with ADHD symptoms, sensation seeking, and impulsivity [17,30]. As the research on the so-called “dark triad” of personality traits revealed that subclinical narcissism is closely related to Machiavellianism and subclinical psychopathy [31], manipulative, opportunistic, and antisocial behaviors might be potential personality features of PCE users as well.

The primary goal of the present study was, therefore, a broad characterization of recently abstinent PCE users regarding their cognitive, behavioral, and personality profile. Based on previous studies showing a higher prevalence of PCE in students with lower grades [1,16,17] and demonstrating cognitive impairment in cocaine and other stimulant drug users [18–20,22], we hypothesized that MPH misuse for PCE is associated with lower cognitive performance. Moreover, in PCE users we expected an increase in psychopathological loads and a specific personality structure similar to recreational stimulant users [23,25,32]. Specifically, we expected that PCE users show more pronounced narcissistic, opportunistic, Machiavellian, and impulsive facets, and less prominent sociable and prosocial behaviors.

## Methods

### Participants

Participants were recruited through flyer advertisements at the University of Zurich and the Swiss Federal Institute of Technology in Zurich, internet advertisement, and via e-mail as study participants from an earlier study on PCE had consented to be contacted again [9]. All participants had to pass an initial telephone screening to assess basic eligibility before they were invited for the assessment at the Psychiatric Hospital of the University of Zurich. Recently abstinent PCE users had to meet the following inclusion criteria: regular MPH use explicitly for PCE during the past 6 months and lifetime use of MPH for PCE on at least 25 occasions. Further inclusion criteria for all participants were 20 to 50 years of age and sufficient knowledge of German language. Exclusion criteria for all participants implied the following conditions: 1) severe medical diseases such as cardiovascular diseases, cancer, HIV, hepatitis, and diabetes; 2) present or prior axis-I psychiatric disorder according to DSM-IV; 3) no family history of a severe DSM-IV psychiatric disorder such as schizophrenia, bipolar disorder, or obsessive-compulsive disorder; 4) lifetime history of a neurological disorder such as meningitis, epilepsy, Tourette syndrome, Parkinson’s disease, dementia, and head injury including loss of consciousness for more than 30 sec; 5) lifetime history of heroin use; 6) daily use of cannabis; 7) regular use of prescription drugs with effects on the central nervous system; and 8) use of other illegal drugs not mentioned before on more than 50 occasions. Prior to the testing session, participants had to abstain from MPH and illegal drugs for at least 72 hours and from alcohol for 24 hours. Adherence with these instructions was assessed by urine testing as described before [22]. The study was approved by the Cantonal Ethics Committee of Zurich. All study participants provided informed consent after being fully informed about the study details.

### Drug use

Current and past use of illegal substances and prescription drugs was assessed by a standardized Interview for Psychotropic Drug Consumption considering the date of last use, average quantity (mg, g, tablets, etc.) used weekly, and total lifetime duration of use [33]. Moreover, urine and hair testing revealed objective quantitative results about recent and past drug use. Urine samples were analyzed by liquid chromatography-tandem mass spectrometry (LC-MS/MS) regarding MPH and ritalinic acid (S1 Method) and by a semi-quantitative enzyme multiplied immunoassay method using a Dimension RXL Max (Siemens, Erlangen, Germany) for all other drugs [22]. MPH and illegal drug use during the past 6 months was assessed by 6-cm hair samples analyzed by LC-MS/MS as described in detail in S2 Method [22].

### Cognitive functions

For matching reasons, premorbid verbal intelligence was assessed by the Multiple-Choice Vocabulary Test (MWT-B). The following classical neuropsychological tests were used to assess cognition of PCE users and stimulant-naïve controls: four tests of the Cambridge Neuropsychological Test Automated Battery (CANTAB) were used to test sustained attention (Rapid Visual Processing, RVP), visuo-spatial memory (Paired Associates Learning, PAL), Spatial Working Memory (SWM), and Intra-Extra-Dimensional Set-Shifting (IED); the Letter-Number-Sequencing Task (LNST) was used to test verbal working memory; and the Rey Auditory Verbal Learning Test (RAVLT) was applied to test declarative verbal memory functions. Similar to our previous studies with cocaine users, four main z-scored cognitive domains (attention, working memory, declarative memory, executive functions) were defined and equally integrated in a global cognitive index (GCI, for details regarding the construction of the cognitive domains see S3 Method) [20,22]. Furthermore, the Iowa Gambling Task (IGT) was used to measure decision-making. Points gained in the IGT were converted into Swiss Francs and disbursed to the participants.

### Social cognition, interaction, and function

Social cognitive functions such as cognitive and emotional empathy as well as theory-of-mind (ToM) were assessed with the Multifaceted Empathy Test (MET) and with the Movie for the Assessment of Social Cognition (MASC), respectively. Moreover, the Distribution Game and the Dictator Game tested social decision-making in an interaction paradigm, while the Social Network Questionnaire (SNQ) provided the number of currently available social contacts. Points gained in both interactive games were converted into Swiss Francs and disbursed to the participants. All tests have been described in detail before [24,25].

### Personality and psychiatric symptoms

Psychiatric symptoms and personality disorders were assessed using the ADHD Self-Rating scale (ADHS-SR), the Structured Clinical Interview for DSM-IV Axis I (SCID-I Interview) and Axis II (SCID-II Questionnaire), and the Beck Depression Inventory (BDI). The Barratt Impulsiveness Scale (BIS-11), the Temperament Character Inventory (TCI), and the Machiavellianism questionnaire (MACH-IV) were included to assess personality. Additionally, the Delay Discounting task (DD) was used to assess delay of gratification/reward impulsivity (references to the neuropsychological tasks and all questionnaires are provided in Table A in S1 File).

### Statistical analysis

PCE users were matched with stimulant-naïve controls on the following variables: age, sex, years of education, proportion of students, verbal intelligence, and proportion of smokers. Quantitative data were analyzed by independent t-tests in order to compare PCE users with controls. For qualitative data, Chi^2^-tests were applied. Person’s product moment correlations were used to evaluate the association between MPH use, cognitive performance, and personality scores and to explore inter-correlations between variables with significant group differences. All statistical analyses were conducted using SPSS Statistics 22 (Dynelytics, Zurich, Switzerland). For group comparisons, *p* < 0.050 was set as the significance level, while for correlation analyses the significance threshold was set at *p* < 0.010 in order to avoid an accumulation of alpha-error.

## Results

**Fig 1:**
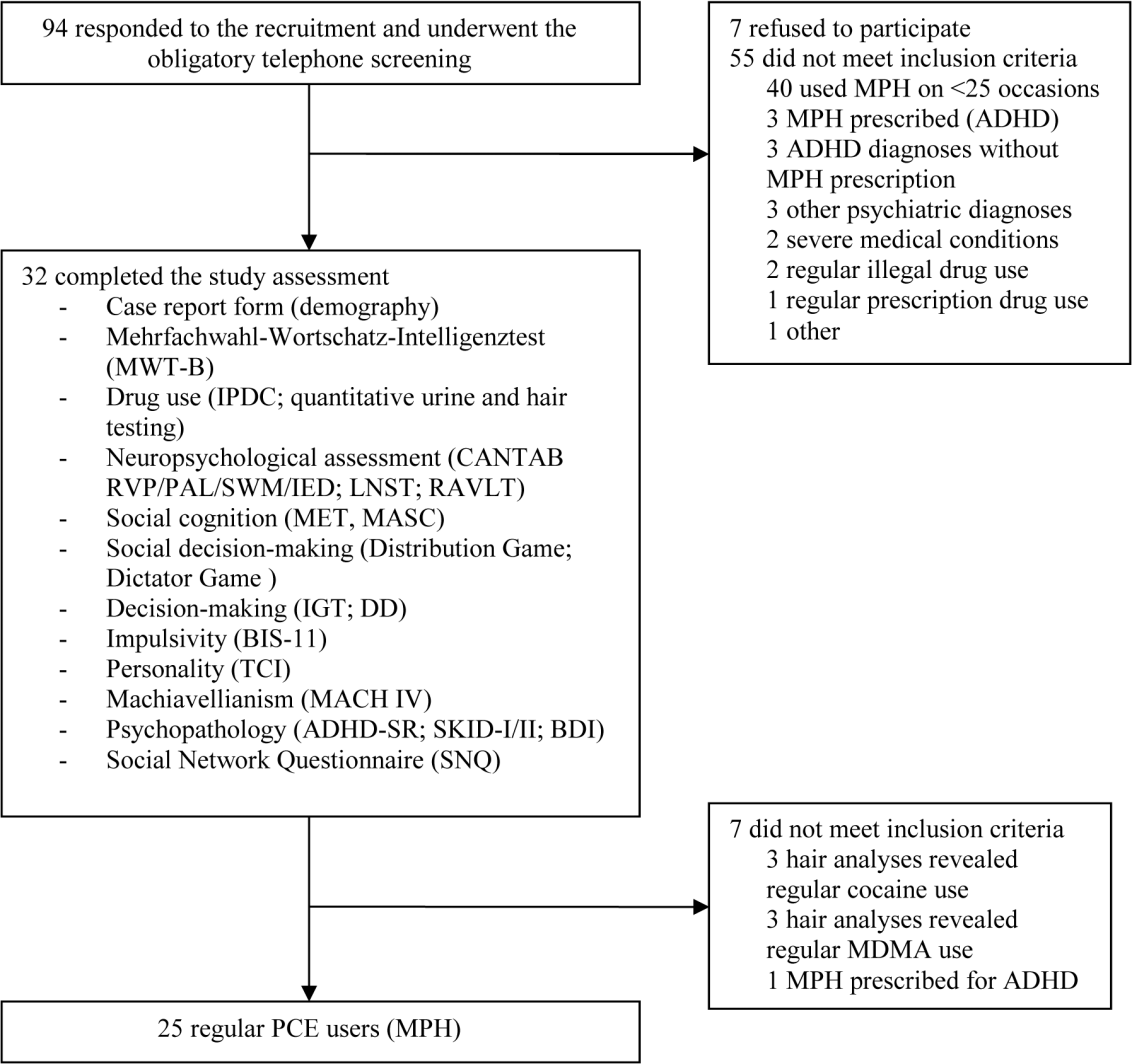
Trial profile. ADHD: Attention Deficit Hyperactivity Disorder, ADHD-SR: Attention Deficit Hyperactivity Disorder Self-Rating scale, BIS-11: Barratt Impulsiveness Scale-11, CANTAB: Cambridge Neuropsychological Test Automated Battery, DD: Delay Discounting task, IDPC: Standardized Interview for Psychotropic Drug Consumption (self-report), IED: Intra-Extra Dimensional Set-Shifting, IGT: Iowa Gambling Task, LNST: Letter Number Sequencing Task, MASC: Movie for the Assessment of Social Cognition, MET: Multifaceted Empathy Test, MDMA: 3,4-Methylendioxy-N-methylamphetamin, MPH: methylphenidate, PAL: Paired Associates Learning, PCE: pharmacological cognitive enhancement, RAVLT: Rey Auditory Verbal Learning Test, RVP: Rapid Visual Information Processing, SCID I/II: Structural Clinical Interview for DSM-IV Axis I/II Disorders, SWM: Spatial Working Memory, TCI: Temperament and Character Inventory.

Ninety-four PCE users contacted us and showed interest in study participation. After a careful telephone screening, we were able to test 32 regular PCE users but only 25 of them met all inclusion criteria and were included in the final analyses (the trial profile is shown in Fig 1). PCE users were matched with 39 stimulant-naïve controls that were tested in the same way. As intended by the matching procedure both groups did not differ regarding age, sex, student status, years of education, verbal IQ, and smoking status, but PCE users reported significantly more ADHD symptoms than controls (Table 1). According to the cut-off of the ADHS-SR questionnaire, five PCE users potentially met the DSM-IV criteria for ADHD but have not been diagnosed with this disorder before and were thus included in the study.

**Table 1:** Demographic characteristics and drug use of stimulant-naïve healthy controls and individuals using methylphenidate for the purpose of pharmacological cognitive enhancement (PCE).

	Controls (*n* = 39)	PCE users (*n* = 25)	*X* ^*2*^ / *t*-test	*df*	*p* value
*Demographics*					
Age	26.2 (5.4)	24.0 (3.0)	1.185	62	0.072
Women	18 (46%)	11 (44%)	0.029	1	0.866
Smoking status (yes)	24 (46%)	15 (44%)	0.029	1	0.866
Student status (yes)	26 (67%)	20 (80%)	1.340	1	0.247
Years of education	11.6 (1.5)	12.0 (1.0)	-1.161	62	0.250
Verbal IQ (MWT-B)	106.0 (8.6)	104.8 (10.5)	0.507	62	0.614
ADHD-SR (range 0–54)	7.3 (5.1)	12.9 (8.5)	-3.303	62	**0**.**002**
BDI sum score	3.5 (4.2)	4.8 (5.2)	-1.029	62	0.308
*Drug use*					
Methylphenidate					
Tablets per week (10mg)	0	2.5 (3.2)			
Years of use	0	2.8 (1.5)			
Cumulative dose (tablets)	0	485.6 (1044.4)			
Last consumption (days)	NA	40.5 (52.2), *n* = 24			
Hair analysis (pq/mg)	0	84.2 (199.1)			
Alcohol					
Grams per week	90.6 (77.0)	92.8 (76.0)	-0.114	62	0.910
Years of use	8.6 (5)	7.2 (3.5)	1.309	62	0.195
Tobacco					
Cigarettes per day	5.6 (8.0)	4.8 (6.4)	0.456	62	0.650
Years of use	5.9 (6.3)	3.7 (3.6)	1.557	62	0.125
Cannabis					
Grams per week	0.2 (0.7)	0.2 (0.4)	0.570	62	0.571
Years of use	3.7 (4.4)	3.3 (3.5)	0.385	62	0.702
Cumulative dose (grams)	965.4 (4423.9)	101.3 (164.0)	0.973	62	0.334
Last consumptions (days)	740.6 (1735.0), *n* = 24	23.5 (24.3), *n* = 13	1.480	35	0.148
Positive urine testing^a^	4 (10%)	2 (8%) ^a^	0.064	1	0.801
Cocaine					
Grams per week	0	0.1 (0.2)	-1.566	62	0.122
Years of use	0 (0)	1.1 (2.8)	-2.499	62	0.**015**
Cumulative dose (grams)	0.2 (0.9)	15.8 (60.4)	-1.620	62	0.110
Last consumptions (days)	1104.8 (947.9), *n* = 3	319.2 (326.4), *n* = 9	2.290	10	**0**.**045**
Positive urine testing^a^	0	0			
Positive hair testing^a^	0	1 (4%)	1.585	1	0.280
Amphetamine					
Grams per week	0	0.01 (0.02)	-1.718	62	0.091
Years of use	0 (0)	0.4 (1.3)	-2.064	62	0.**043**
Cumulative dose (grams)	0.003 (0.02)	0.6 (2.4)	-1.441	62	0.155
Last consumptions (days)	547.2 (258.0), *n* = 2	346.4 (724.9), *n* = 6	0.367	6	0.726
Positive urine testing^a^	0	1 (4%)	1.651	1	0.199
Positive hair testing^a^	0	1 (4%)	1.585	1	0.280
MDMA					
Tablets per week	0	0.04 (0.2)	-1.399	62	0.167
Years of use	0 (0)	0.4 (1.0)	-2.342	62	0.**022**
Cumulative dose (tablets)	0.13 (0.4)	3.4 (9.0)	-2.256	62	**0**.**028**
Last consumption (days)	3100.8 (1289.8), *n* = 2	31.3 (26.0), *n* = 3	3.976	6	**0**.**007**
Positive hair testing^a^	0	2 (8%)	3.221	1	0.073

Data are means and standard deviations, or number and percent. Significant p-values are shown in bold.

^a^For cut-offs see S1 and S2 Methods.

ADHD-SR: Attention Deficit Hyperactivity Disorder Self-Rating scale, BDI: Beck Depression Inventory, IQ: intelligence quotient, MWT-B: Mehrfachwahl-Wortschatz-Test (vocabulary test), PCE: pharmacological cognitive enhancement.

On average, PCE users reported the intake of MPH for PCE since 2.8 years, used 2.5 tablets with 10mg MPH per week, have taken 486 MPH tablets in their lifetime, and were abstinent from MPH since 41 days (Table 1). Six PCE users featured positive urine testing for ritalinic acid (mean 248ng/ml, range 3–1312 ng/ml), while only one PCE user revealed small traces of MPH (14 ng/ml). Self-reported weekly MPH use was significantly correlated with hair concentrations of MPH over the past 6 months (*r* = 0.640, *p* < 0.001, *n* = 25).

**Fig 2:**
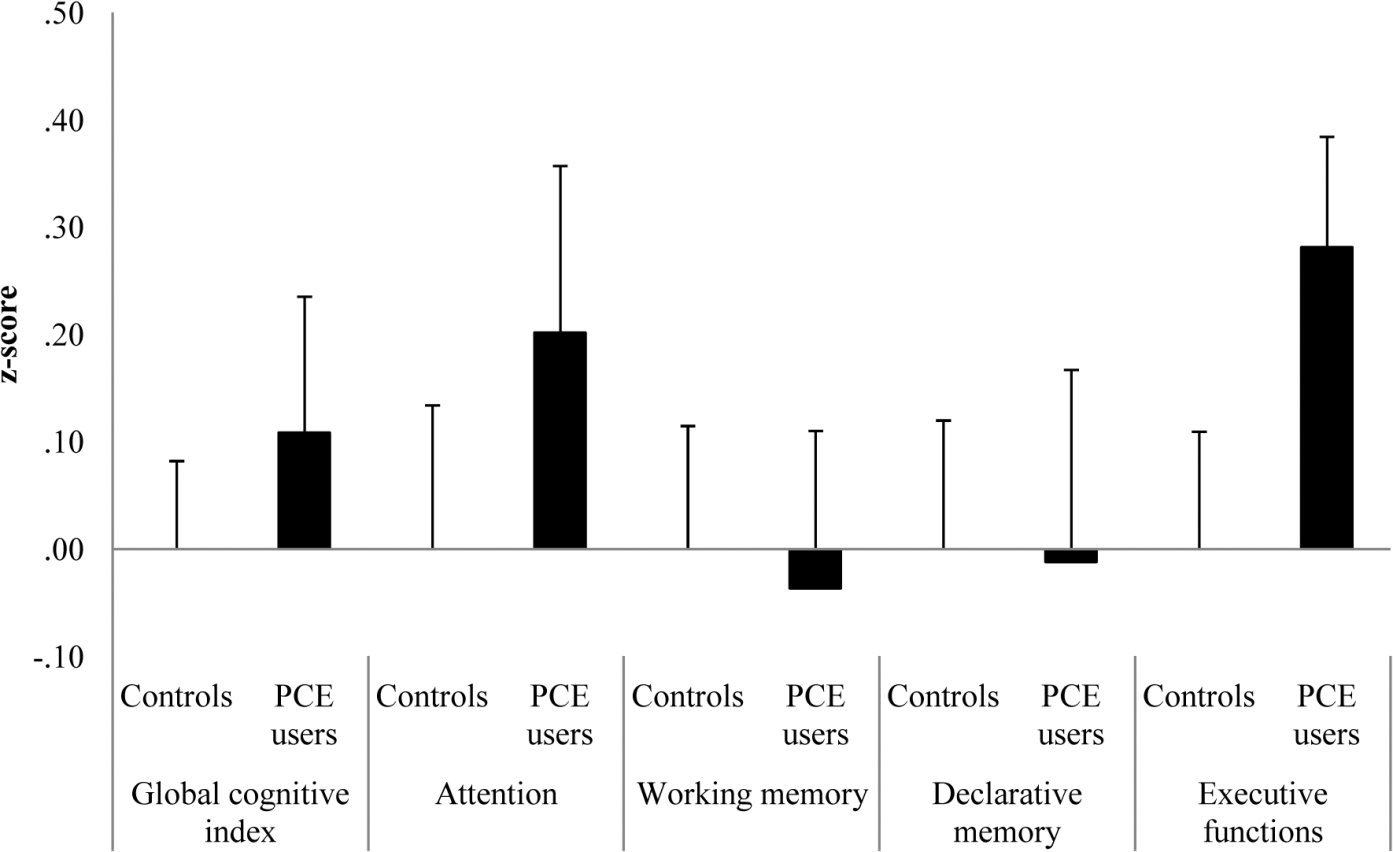
Mean z-scores and standard errors of means for the global cognitive index (GCI) and four cognitive domains.

**Fig 3:**
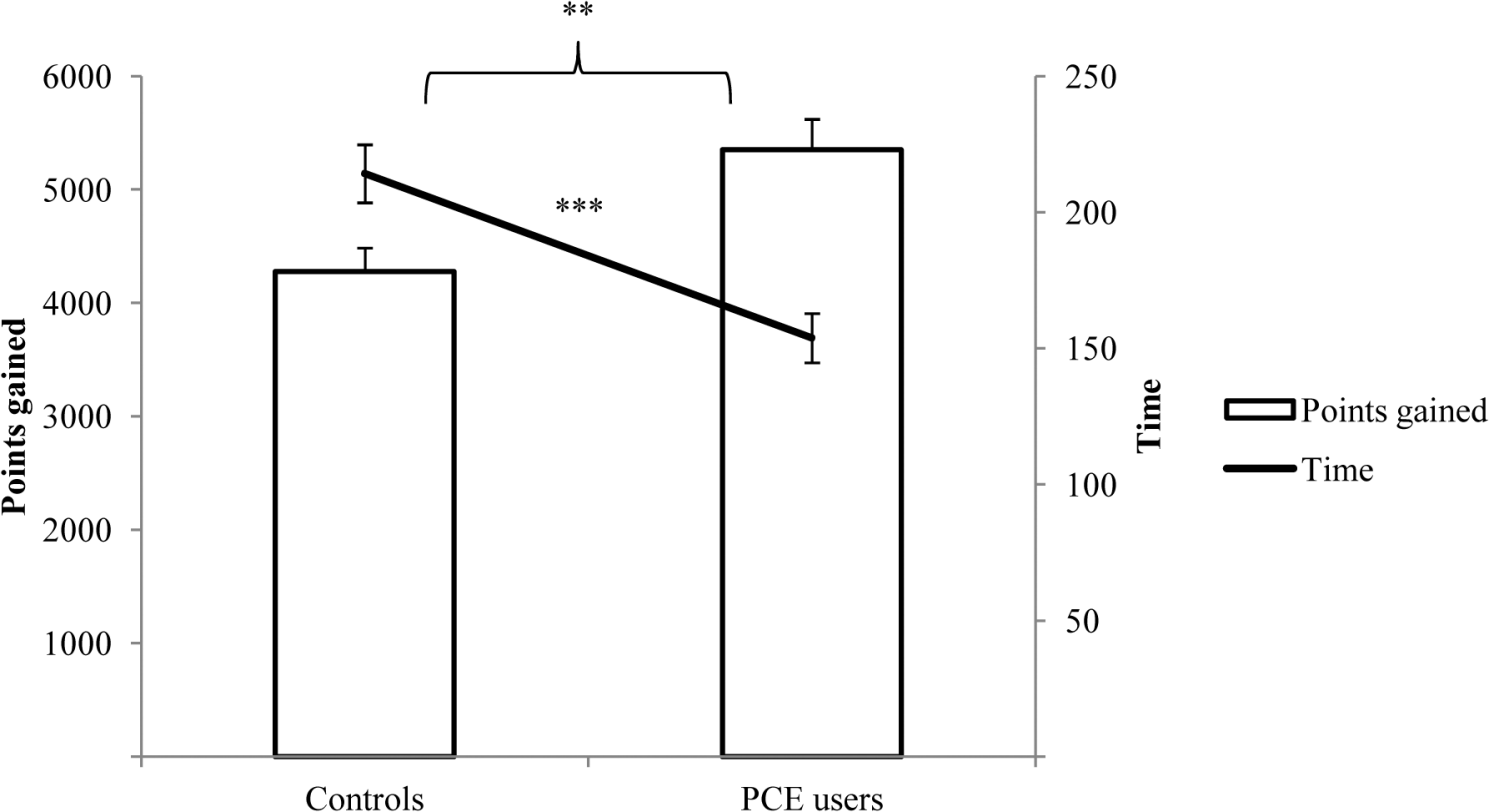
Means and standard error of means of points gained and of processing time (sec) in the Iowa Gambling Task (IGT). ** *p*< 0.010, and ***p < 0.001.

PCE users showed no significant differences in the four cognitive domains and the GCI compared to controls (Fig 2). However, the executive functions showed a moderate effect size (*d* = 0.44) with regard to a superior performance of the PCE users, which was mainly explained by a significantly better performance in the strategy subscore of the SWM (S1 Table). In the IGT, PCE users gained more points in a shorter time (Fig 3), showing better performance in the second and in the fourth quartile (S1 Fig).

PCE users displayed higher levels of novelty seeking (mainly explained by significantly higher disorderliness) and revealed lower scores in social reward dependence (primarily due to significantly lower sentimentality) compared to controls. No group differences were found for delay discounting, but PCE users showed elevated self-reported impulsivity in the BIS-11 (specifically in the attention subscores), higher negativistic and antisocial personality traits in the SCID-II questionnaire, as well as higher Machiavellianism in the MACH-IV (Table 2). Compared to controls, PCE users reported significantly fewer social contacts and their decisions in the social interaction tasks were more self-serving as they gave less money to the opposite player B while preferring higher payoffs for themselves (Table 2).

**Table 2:** Personality traits and social cognition and interaction of stimulant-naïve healthy controls and individuals using methylphenidate for the purpose of pharmacological cognitive enhancement (PCE).

	Controls (*n*· = ·39)	PCE users (*n*· = ·25)	*t*-test	*df*	*p* value	Cohen's *d*
*Personality*						
BIS-11 sum score	61.6 (8.4)	66.8 (11.0)	-2.145	62	**0.036**	0.53
BIS-11 Motor impulsiveness	21.7 (3.1)	23.9 (6.0)	-1.941	62	0.057	0.49
BIS-11 Nonplanning impulsiveness	25.4 (4.2)	26.2 (5.1)	-0.690	62	0.493	0.18
BIS-11 Attentional impulsiveness	14.6 (3.2)	16.8 (4.4)	-2.293	62	**0.025**	0.57
BIS-11 Attention	9.5 (2.3)	11.1 (2.8)	-2.428	62	**0.018**	0.60
BIS-11 Cognitive Inflexibility	5.1 (1.7)	14.2 (3.3)	-1.284	62	0.204	0.33
TCI Novelty Seeking	21.8 (5.2)	24.6 (5.9)	-2.003	62	**0**.**050**	0.50
TCI Exploratory excitability	7.9 (2.0)	8.3 (2.5)	-0.804	62	0.425	0.21
TCI Impulsiveness	4.3 (2.1)	4.6 (2.2)	-0.563	62	0.576	0.14
TCI Extravagance	5.4 (1.8)	5.9 (1.9)	-0.946	62	0.348	0.24
TCI Disorderliness	4.2 (1.8)	5.8 (1.7)	-3.566	62	**0**.**001**	0.84
TCI Harm avoidance	14.3 (5.6)	13.2 (6.9)	0.747	62	0.458	0.19
TCI Reward Dependence	16.9 (4.0)	14.6 (4.2)	2.182	62	**0**.**033**	0.54
TCI Sentimentality	6.5 (1.8)	5.4 (1.8)	2.299	62	**0**.**025**	0.57
TCI Attachment	6.5 (1.9)	5.5 (2.4)	1.850	62	0.069	0.47
TCI Dependence	4.0 (1.6)	3.7 (1.6)	0.565	62	0.574	0.15
TCI Persistence	4.3 (2.1)	3.4 (2.1)	1.681	62	0.098	0.42
TCI Self-Directedness	33.9 (6.0)	31.2 (6.4)	1.704	62	0.093	0.43
TCI Cooperativeness	33.5 (5.7)	31.5 (5.0)	1.375	62	0.174	0.35
TCI Self-Transcendence	10.2 (5.2)	9.2 (6.6)	0.695	62	0.490	0.18
SCID-II Avoidant	1.2 (1.4)	0.7 (1.1)	1.526	62	0.132	0.39
SCID-II Dependent	1.2 (1.1)	1.2 (1.0)	-0.035	62	0.973	0.01
SCID-II Obsessive-compulsive	3.5 (1.8)	3.6 (1.6)	-0.256	62	0.799	0.07
SCID-II Negativistic	1.2 (1.4)	2.0 (1.4)	-2.486	62	**0**.**016**	0.61
SCID-II Depressive	1.1 (1.6)	1.4 (1.7)	-0.559	62	0.578	0.14
SCID-II Paranoid	1.6 (1.8)	1.8 (1.9)	-0.359	62	0.721	0.09
SCID-II Schizotypal	1.3 (1.6)	1.1 (1.0)	0.628	62	0.532	0.16
SCID-II Schizoid	0.7 (1.1)	1.2 (1.3)	-1.699	62	0.094	0.43
SCID-II Histrionic	1.9 (1.5)	2.5 (1.9)	-1.343	62	0.184	0.34
SCID-II Narcissistic	2.4 (2.5)	3.2 (2.3)	-1.241	62	0.219	0.32
SCID-II Borderline	2.4 (2.1)	2.3 (2.0)	0.303	62	0.763	0.08
SCID-II Antisocial	1.9 (1.6)	3.0 (2.7)	-2.011	62	**0**.**049**	0.50
*Social cognition and interaction*						
MACH-IV sum score	89.7 (12.3)	97.0 (10.5)	-2.561	61	**0**.**013**	0.64
DD *k* parameter all	0.012 (0.018)	0.022 (0.032)	-1.615	62	0.111	0.41
MET Direct Empathy	5.1 (1.2)	4.7 (1.2)	1.426	62	0.159	0.36
MET Indirect Empathy	4.8 (1.2)	4.6 (1.3)	0.420	62	0.676	0.11
MET Cognitive Empathy	25.4 (3.8)	23.0 (4.9)	2.273	62	**0**.**026**	0.56
MASC Total ToM errors	10.2 (4.6)	8.2 (3.2)	1.905	62	0.061	0.48
Social network size (SNQ)	21.5 (7.3)	17.8 (5.3)	2.213	62	**0.031**	0.55
Distribution game, payoff player B	19.0 (8.0)	17.0 (9.6)	0.882	62	0.381	0.23
Dictator game, payoff player B	16.6 (12.2)	10.3 (9.4)	2.196	62	**0.032**	0.55

Data are means and standard deviations. Significant p-values are shown in bold. BIS: Barratt Impulsiveness Scale, DD: Delay Discounting task, MACH-IV: Machiavellianism Scale, MASC: Movie for the Assessment of Social Cognition, MET: Multifaceted Empathy Test, PCE: pharmacological cognitive enhancement, SCID-II: Structural Clinical Interview for DSM-IV Axis-II Disorders, SNQ: Social network questionnaire, TCI: Temperament and Character Inventory, ToM: Theory-of-Mind.

When assessing mental perspective-taking (ToM), PCE users made somewhat fewer errors in the MASC but the difference was not significant. Although PCE users revealed a slightly enhanced mental perspective-taking in the MASC, they showed, however, significantly lower cognitive empathy in the MET, indicating worse emotion recognition from complex picture material (Table 2).

Neither cognitive performance nor personality scales were correlated with any MPH consumption parameters, indicating that the shown abnormalities of PCE users are likely not drug-induced. Machiavellianism was positively correlated with the TCI novelty seeking subscore disorderliness and the SCID-II negativistic score but negatively correlated with TCI social reward dependence and its subscore sentimentality. Not surprisingly, the ADHS-SR score was highly correlated with several BIS-11 scores but also with the SCID-II negativistic score. TCI disorderliness and the SCID-II antisocial score were positively correlated as well (S2 Table). These correlations reflect overlapping concepts of impulsivity and sociability as measured by the different questionnaires.

## Discussion

The aim of the study was to investigate whether regular PCE users show impaired cognitive functions and a specific pattern of personality traits. The study revealed two main findings: 1) recently abstinent PCE users and stimulant-naïve controls performed equally in most of the cognitive tasks but PCE users performed better in strategic thinking and decision-making, and 2) PCE users showed higher impulsivity, higher novelty seeking, higher Machiavellianism, and more pronounced antisocial and negativistic personality traits, in combination with lower social reward dependence compared to controls. In line with this personality pattern, they behaved more opportunistically in social interaction tasks, showed less cognitive empathy, and reported having a smaller social network. Importantly, these results cannot be explained by withdrawal effects as the mean abstinence duration from MPH was 41 days and only one subject has shown very small traces of MPH in the urine testing.

The finding that regular PCE users showed elevated attentional impulsivity but no cognitive impairment might indicate their motivation to use MPH for PCE. As it was shown that only individuals with low baseline performance show cognitive improvements using stimulant drugs [34], it is unlikely that MPH actually improved general cognitive functioning of the present PCE users because they already performed very well and sometimes better than controls. However, MPH is effective to treat symptoms of ADHD such as attentional impulsivity [10]. In fact, PCE users in the present study showed more ADHD symptoms and a previous study found procognitive effects of MPH specifically in healthy individuals with high impulsivity [35]. Thus, MPH might improve impulse control of PCE users, helping them to begin and sustain studying, rather than enhancing cognition directly. Consequently, not everyone benefits from MPH use and opposite cognitive effects (improvement and impairment) of the same MPH dose might even occur in the same individual depending on task requirements [10,13]. As a specific predisposition such as high impulsivity is needed to benefit from MPH use and not everyone is willing to use PCE anyway [29], a forthcoming epidemic of MPH use for PCE is considered unlikely.

Although the groups did not significantly differ on the SCID-II narcissistic scale as initially hypothesized, PCE users showed more negativistic and antisocial personality traits, and higher Machiavellianism compared to controls. Interestingly, the SCID-II narcissistic subscale was significantly correlated with Machiavellianism (*r·* = ·0.38, *p·*<·0.010) and the SCID-II negativistic subscale (*r·* = ·0.51, *p·*<·0.001), confirming that narcissism, negativism, and Machiavellianism show a considerable phenomenological overlap. Thus, PCE users showed a specific pattern of personality traits that has been conceptualized as the “dark triad” [31]. Moreover, with their increased novelty seeking, higher impulsivity, and antisocial tendencies, PCE users share a number of personality features with recreational stimulant users [23,36]. Additionally, PCE users behaved less prosocial in a money distribution game similar to recreational and dependent cocaine users as shown recently [25]. As intensity of cocaine use was not correlated with social decision-making, Hulka et al. suggested that the opportunistic behavior of stimulant users might be a stable trait and possibly a predisposition for the initiation of stimulant use [25]. Furthermore, similar to cocaine users, PCE users also displayed a smaller social network than controls [24]. This might be explained by the fact that PCE users are less sociable (as their personality profile suggests) and, thus, less integrated in social networks. Additionally, the smaller social network might mirror an intensified cost-benefit thinking of PCE users, and a more strategic selection of friends as supported by the present findings in IGT decision-making and strategic thinking.

Our findings are subject to some limitations. First, the number of PCE users was relatively small. This is obviously a threat to the statistical power of the reported analysis but, at the same time, a further implicit result of the study. In fact, it was hard to find individuals with regular MPH use for PCE who reported no concurrent regular use of other illegal drugs and no ADHD diagnosis. Second, the fact that the data were restricted to PCE users, who used MPH without regular co-use of illegal drugs of abuse, is a further limitation as it was shown previously that PCE users show a higher prevalence of illegal drug use compared to non-users [9,17]. Therefore, the question arises, whether we tested only a very unique group within the already very specific group of PCE users. Nevertheless, the exclusion of PCE users with regular illegal drug use was inevitable in order to explain differences between PCE users and controls exclusively by the MPH use. Moreover, previous research revealed that PCE occurs most likely during short periods of exam preparation and daily or high dose use of PCE is rare [9]. Thus, through our inclusion criteria, we likely skimmed only the most intense PCE users. Third, we used a cross-sectional design but a longitudinal design would have been most appropriate to investigate cause-effect relationships between PCE drug use and changes in cognition and personality.

### Conclusion

This is the first study that broadly characterized individuals regularly using MPH for PCE by applying a comprehensive neuropsychological test battery in combination with a thorough personality assessment and urine and hair testing. Our findings indicate that the regular nonmedical MPH use for PCE over more than two years was not associated with cognitive deficits. PCE users performed equally to controls, or even better in tasks requiring strategic thinking, which disproves the assumption that PCE is a compensation for cognitive deficits [1,16,17]. As the personality profile of PCE users shared some features with recreational illegal stimulant users, such as higher novelty seeking and impulsivity, we propose instead that PCE users may aim to improve their impulse control in order to optimize their own learning compliance. PCE users were also found to be less prosocial, less emphatic, and more Machiavellianism, which is in line with their enhanced strategic thinking and planning behavior. Thus, PCE users may instrumentalize MPH as little helper [37] in order to maximize their own benefits. Finally, the overall personality profile of PCE users is highly specific disproving the often made assumption that PCE will widely spread in society.

## Supporting Information

S1 Fig Means and standard errors for quartiles (Q1-Q4) and the net score (good minus bad cards) in the Iowa Gambling Task; *p < 0.050, PCE: pharmacological cognitive enhancement. (TIF)

S1 File Neuropsychological Assessment. References to neuropsychological tasks, interviews, and questionnaires used (Table A).
(DOCX)

S1 Method Urine testing. (DOCX)

S2 Method Hair testing. (DOCX)

S3 Method Construction of the four cognitive domain scores. Significant partial correlations with a *p*-level below 1% are shown and marked as: **p < 0.010, ***p < 0.001. ADHD-SR: ADHD Self-Rating Scale, BIS: Barratt Impulsiveness Scale, MET: Multifaceted Empathy Test, NS: Novelty Seeking, PCE: pharmacological cognitive enhancement, RD: Reward Dependence, SCID I/II: Structural Clinical Interview for DSM-IV Axis I/II Disorders, SNQ: Social Network Questionnaire, SWM: Spatial Working Memory, TCI: Temperament and Character Inventory.
(DOCX)

S1 Table Global cognitive index (GCI), the four cognitive domain z-scores, and neuropsychological test scores of stimulant-naïve healthy controls and individuals using methylphenidate for the purpose of pharmacological cognitive enhancement (PCE). Data are means and standard deviations. Significant p-values are shown in bold. IED: Intra-Extra Dimensional Set-Shifting, LNST: Letter Number Sequencing Task, PAL: Paired Associates Learning, PCE: pharmacological cognitive enhancement, RAVLT: Rey Auditory Verbal Learning Test, RVP: Rapid Visual Information Processing, SWM: Spatial Working Memory.
(DOCX)

S2 Table Pearson’s product-moment correlations between test outcomes and clinical measures of social functioning with significant group differences between stimulant-naïve healthy controls (n = 39) and pharmacological cognitive enhancement users (*n* = 25). Significant partial correlations with a *p*-level below 1% are shown and marked as: **p < 0.010, ***p < 0.001. ADHD-SR: ADHD Self-Rating Scale, BIS: Barratt Impulsiveness Scale, MET: Multifaceted Empathy Test, NS: Novelty Seeking, PCE: pharmacological cognitive enhancement, RD: Reward Dependence, SCID I/II: Structural Clinical Interview for DSM-IV Axis I/II Disorders, SNQ: Social Network Questionnaire, SWM: Spatial Working Memory, TCI: Temperament and Character Inventory.
(DOCX)

## References

[1] McCabe SE, Knight JR, Teter CJ, Wechsler H. Non-medical use of prescription stimulants among US college students: prevalence and correlates from a national survey. Addiction. 2005;99:96–106. 10.1111/j.1360-0443.2005.00944.x 15598197

[2] Greely HT, Sahakian BJ, Harris J, Kessler RC, Gazzaniga M, Campbell P, et al Towards responsible use of cognitive-enhancing drugs by the healthy. Nature. 2008;456:702–5. 10.1038/456702a 19060880

[3] Farah MJ, Illes J, Cook-Deegan R, Gardner H, Kandel E, King P, et al Neurocognitive enhancement: what can we do and what should we do? Nat Rev Neurosci. 2004;5:421–5. 15100724 10.1038/nrn1390

[4] Bostrom N, Sandberg A. Cognitive enhancement: methods, ethics, regulatory challenges. Sci Eng Ethics. 2009;15:311–41. 10.1007/s11948-009-9142-5 19543814

[5] Morris K. Experts urge smart thinking on cognitive enhancers. Lancet Neurol. 2008;7:476–7. 10.1016/S1474-4422(08)70101-7 18485312

[6] Bogle KE, Smith BH. Illicit methylphenidate use: a review of prevalence, availability, pharmacology, and consequences. Curr Drug Abuse Rev. 2009;2:157–76. 19630746 10.2174/1874473710902020157

[7] McCabe SE, West BT, Teter CJ, Boyd CJ. Trends in medical use, diversion, and nonmedical use of prescription medications among college students from 2003 to 2013: Connecting the dots. Addict Behav. 2014;39:1176–82. 10.1016/j.addbeh.2014.03.008 24727278 PMC4349373

[8] Maier LJ, Schaub MP. The use of prescription drugs and drugs of abuse for neuroenhancement in Europe: Not widespread but a reality. Eur Psychol. in press; 10.1027/1016-9040/a000228

[9] Maier LJ, Liechti ME, Herzig F, Schaub MP. To Dope or Not to Dope: Neuroenhancement with Prescription Drugs and Drugs of Abuse among Swiss University Students. PLoS One. 2013;8:e77967 10.1371/journal.pone.0077967 24236008 PMC3827185

[10] Wood S, Sage JR, Shuman T, Anagnostaras SG. Psychostimulants and cognition: a continuum of behavioral and cognitive activation. Pharmacol Rev. 2014;66:193–221. 10.1124/pr.112.007054 24344115 PMC3880463

[11] Repantis D. Psychopharmacological Neuroenhancement: Evidence on Safety and Efficacy In: Hildt E, Franke AG, editors. Cognitive Enhancement: An Interdisciplinary Perspective. Dordrecht: Springer Netherlands; 2013.

[12] Husain M, Mehta MA. Cognitive enhancement by drugs in health and disease. Trends Cogn Sci. 2011;15:28–36. 10.1016/j.tics.2010.11.002 21146447 PMC3020278

[13] Van der Schaaf ME, Fallon SJ, Ter Huurne N, Buitelaar J, Cools R. Working memory capacity predicts effects of methylphenidate on reversal learning. Neuropsychopharmacology. 2013;38:2011–8. 10.1038/npp.2013.100 23612436 PMC3746683

[14] Quednow BB. Ethics of neuroenhancement: a phantom debate. Biosocieties. 2010;5:153–6.

[15] Rubia K, Alegria AA, Cubillo AI, Smith AB, Brammer MJ, Radua J. Effects of Stimulants on Brain Function in Attention-Deficit/Hyperactivity Disorder: A Systematic Review and Meta-Analysis. Biol Psychiatry. 2013;76:616–28. 10.1016/j.biopsych.2013.10.016 24314347 PMC4183380

[16] Franke AG, Bonertz C, Christmann M, Huss M, Fellgiebel A, Hildt E, et al Non-medical use of prescription stimulants and illicit use of stimulants for cognitive enhancement in pupils and students in Germany. Pharmacopsychiatry. 2011;44:60–6. 10.1055/s-0030-1268417 21161883

[17] Rabiner DL, Anastopoulos AD, Costello EJ, Hoyle RH, McCabe SE, Swartzwelder HS. Motives and perceived consequences of nonmedical ADHD medication use by college students: are students treating themselves for attention problems? J Atten Disord. 2009;13:259–70. 10.1177/1087054708320399 18664714

[18] Reske M, Eidt CA, Delis DC, Paulus MP. Nondependent stimulant users of cocaine and prescription amphetamines show verbal learning and memory deficits. Biol Psychiatry. 2010;68:762–9. 10.1016/j.biopsych.2010.04.021 20605137 PMC2949490

[19] Reske M, Delis DC, Paulus MP. Evidence for subtle verbal fluency deficits in occasional stimulant users: quick to play loose with verbal rules. J Psychiatr Res. 2011;45:361–8. 10.1016/j.jpsychires.2010.07.005 20673916 PMC3424267

[20] Vonmoos M, Hulka LM, Preller KH, Minder F, Baumgartner MR, Quednow BB. Cognitive Impairment in Cocaine Users is Drug-Induced but Partially Reversible: Evidence from a Longitudinal Study. Neuropsychopharmacology. 2014;39:2200–10. 10.1038/npp.2014.71 24651468 PMC4104339

[21] Nyberg F. Structural plasticity of the brain to psycho-stimulant use. Neuropharmacology. 2014;87:115–24. 10.1016/j.neuropharm.2014.07.004 25018041

[22] Vonmoos M, Hulka LM, Preller KH, Jenni D, Baumgartner MR, Stohler R, et al Cognitive dysfunctions in recreational and dependent cocaine users: role of attention-deficit hyperactivity disorder, craving and early age at onset. Br J Psychiatry. 2013;203:35–43. 10.1192/bjp.bp.112.118091 23703315

[23] Vonmoos M, Hulka LM, Preller KH, Jenni D, Schulz C, Baumgartner MR, et al Differences in self-reported and behavioral measures of impulsivity in recreational and dependent cocaine users. Drug Alcohol Depend. 2013;133:61–70. 10.1016/j.drugalcdep.2013.05.032 23806872

[24] Preller KH, Hulka LM, Vonmoos M, Jenni D, Baumgartner MR, Seifritz E, et al Impaired emotional empathy and related social network deficits in cocaine users. Addict Biol. 2014;19:452–66. 10.1111/adb.12070 23800218

[25] Hulka LM, Eisenegger C, Preller KH, Vonmoos M, Jenni D, Bendrick K, et al Altered social and non-social decision-making in recreational and dependent cocaine users. Psychol Med. 2014;44:1015–28. 10.1017/S0033291713001839 23870112

[26] Schmid Y, Hysek CM, Simmler LD, Crockett MJ, Quednow BB, Liechti ME. Differential effects of MDMA and methylphenidate on social cognition. J Psychopharmacol. 2014;28:847–56. 10.1177/0269881114542454 25052243

[27] Hysek CM, Simmler LD, Schillinger N, Meyer N, Schmid Y, Donzelli M, et al Pharmacokinetic and pharmacodynamic effects of methylphenidate and MDMA administered alone or in combination. Int J Neuropsychopharmacol. 2014;17:371–81. 10.1017/S1461145713001132 24103254

[28] Schleim S, Quednow BB. Debunking the neuroenhancement debate. In: TerMeulen R, Mohamed AD, Hall W, editors. Rethinking Cognitive Enhancement. Oxford: Oxford University Press; in press.

[29] Sattler S, Mehlkop G, Graeff P, Sauer C. Evaluating the drivers of and obstacles to the willingness to use cognitive enhancement drugs: the influence of drug characteristics, social environment, and personal characteristics. Subst Abuse Treat Prev Policy. 2014;9:8 10.1186/1747-597X-9-8 24484640 PMC3928621

[30] Weyandt LL, Janusis G, Wilson KG, Verdi G, Paquin G, Lopes J, et al Nonmedical prescription stimulant use among a sample of college students: relationship with psychological variables. J Atten Disord. 2009;13:284–96. 10.1177/1087054709342212 19767596

[31] Rauthmann JF, Kolar GP. How “dark” are the Dark Triad traits? Examining the perceived darkness of narcissism, Machiavellianism, and psychopathy. Pers Individ Dif. 2012;53:884–9.

[32] Preller KH, Herdener M, Schilbach L, Stämpfli P, Hulka LM, Vonmoos M, et al Functional changes of the reward system underlie blunted response to social gaze in cocaine users. Proc Natl Acad Sci U S A. 2014;111:2842–7. 10.1073/pnas.1317090111 24449854 PMC3932870

[33] Quednow BB, Kühn K-U, Hoenig K, Maier W, Wagner M. Prepulse inhibition and habituation of acoustic startle response in male MDMA (‘ecstasy') users, cannabis users, and healthy controls. Neuropsychopharmacology. 2004;29:982–90. 14970829 10.1038/sj.npp.1300396

[34] De Jongh R, Bolt I, Schermer M, Olivier B. Botox for the brain: enhancement of cognition, mood and pro-social behavior and blunting of unwanted memories. Neurosci Biobehav Rev. 2008;32:760–76. 10.1016/j.neubiorev.2007.12.001 18295885

[35] Clatworthy PL, Lewis SJG, Brichard L, Hong YT, Izquierdo D, Clark L, et al Dopamine release in dissociable striatal subregions predicts the different effects of oral methylphenidate on reversal learning and spatial working memory. J Neurosci. 2009;29:4690–6. 10.1523/JNEUROSCI.3266-08.2009 19369539 PMC6665353

[36] Rounsaville BJ. Treatment of cocaine dependence and depression. Biol Psychiatry. 2004;56:803–9. 15556126 10.1016/j.biopsych.2004.05.009

[37] Sahakian BJ, Morein-Zamir S. Professor’s little helper. Nature. 2007;450:1157–9. 18097378 10.1038/4501157a

