# Supplementary figures and images for: Pharmacological Cognitive Enhancement in Healthy Individuals: A Compensation for Cognitive Deficits or a Question of Personality?
Source: PLoS One. 2015 Jun 24;10(6):e0129805. doi: 10.1371/journal.pone.0129805 (PMC4479570; doi:10.1371/journal.pone.0129805)

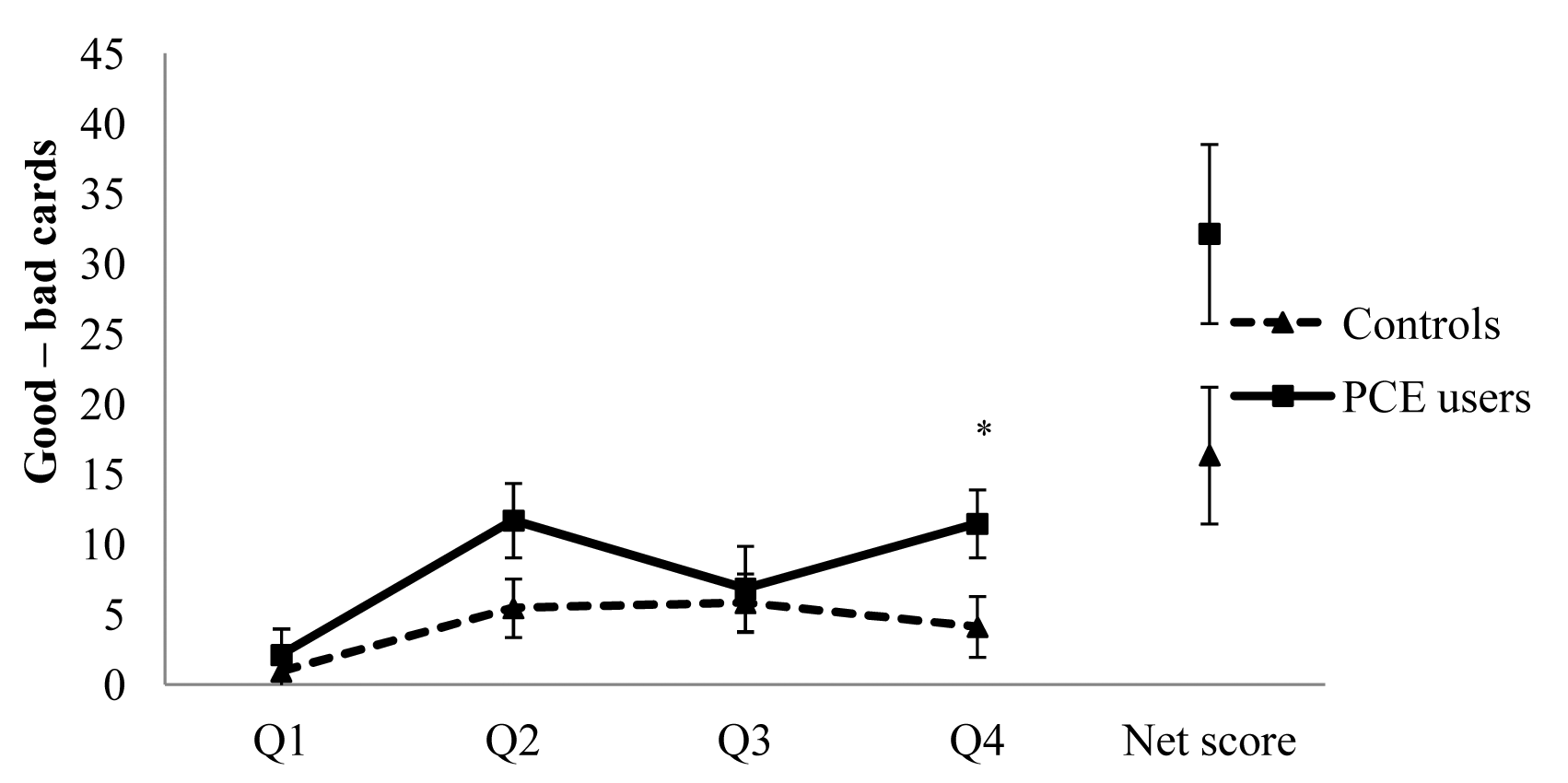

Supplement: S1 Fig — (TIF) [file pone.0129805.s001.tif]
