# Supplementary material for: Pharmacological Cognitive Enhancement in Healthy Individuals: A Compensation for Cognitive Deficits or a Question of Personality?
Source: PLoS One. 2015 Jun 24;10(6):e0129805. doi: 10.1371/journal.pone.0129805 (PMC4479570; doi:10.1371/journal.pone.0129805)
Supplement: S1 File — References to neuropsychological tasks, interviews, and questionnaires used (Table A). (DOCX) [file pone.0129805.s002.docx]

| **Test abbreviation** | **Test name** | **Reference** |
| --- | --- | --- |
| **Drug use** |  |  |
| IPDC | Interview for Psychotropic Drug Consumption | Quednow et al. (2004) [1] |
| **Cognition** | | |
| MWT-B | Mehrfach-Wortwahl-Intelligenztest | Lehrl (1989) [2] |
| CANTAB | Cambridge Neuropsychological Test Automated Battery | Strauss et al. (2006) [3] |
| RVP | Rapid Visual Processing | Jones (1992) [4] |
| PAL | Paired Associates Learning | Sahakian et al. (1988) [5] |
| SWM | Spatial Working Memory | Morris et al. (1988) [6] |
| IED | Intra-Extra-Dimensional Set Shifting | Downes et al. (1989) [7] |
| LNST | Letter Number Sequencing Task | Wechsler (1997) [8] |
| RAVLT | Rey Auditory Verbal Learning Test | Rey (1964)[9]; Helmstaedter et al.(2001) [10] |
| IGT | Iowa Gambling Task | Bechara et al. (2002) [11] |
| **Social cognition, interaction, and function** | | |
| MET | Multifaceted Empathy Test | Dziobek (2008) [12] |
| MASC | Movie for the Assessment of Social Cognition | Dziobek (2006) [13] |
| SNQ | Social Network Questionnaire | Linden et al. (2007) [14] |
| Distribution Game | Distribution Game | Engelmann & Strobel (2004) [15] |
| Dictator Game | Dictator Game | Charness & Rabin (2002) [16] |
| **Personality and psychiatric symptoms** | | |
| ADHS-SR | ADHD Self-Rating scale | Rösler et al. (2004) [17] |
| SCID-I | Structured Clinical Interview for DSM-IV Axis I | Wittchen et al. (1997) [18] |
| SCID-II | Structured Clinical Interview for DSM-IV Axis II | Wittchen et al. (1997) [19] |
| BDI | Beck Depression Inventory | Beck et al. (1988) [20] |
| BIS-11 | Barratt Impulsiveness Scale | Patton et al. (1995) [21] |
| TCI | Temperament Character Inventory | Cloninger et al. (1994) [22]; Berth et al. (2001) [23] |
| MACH-IV | Machiavellianism Test | Christie & Geis (1970) [24] |
| DD | Delay Discounting Task | Kirby et al. (2004) [25] |

**S1 File. Neuropsychological Assessment.**

**Table A. References to neuropsychological tasks, interviews, and questionnaires use**

**References**

1. Quednow BB, Kühn K-U, Hoenig K, Maier W, Wagner M. Prepulse inhibition and habituation of acoustic startle response in male MDMA (’ecstasy') users, cannabis users, and healthy controls. Neuropsychopharmacology. 2004;29:982–90.

2. Lehrl S. Mehrfachwahl-Wortschatz-Intelligenztest: MWT-B. 1989.

3. Strauss E, Sherman EMS, Spreen O. A Compendium of Neuropsychological Tests: Administration, Norms, and Commentary. 2006.

4. Jones GM, Sahakian BJ, Levy R, Warburton DM, Gray JA. Effects of acute subcutaneous nicotine on attention, information processing and short-term memory in Alzheimer’s disease. Psychopharmacology. 1992 p. 485–94.

5. Sahakian BJ, Morris RG, Evenden JL, Heald A, Levy R, Philpot M, et al. A comparative study of visuospatial memory and learning in Alzheimer-type dementia and Parkinson’s disease. Brain. 1988;111:695–718.

6. Morris RG, Downes JJ, Sahakian BJ, Evenden JL, Heald A, Robbins TW. Planning and spatial working memory in Parkinson’s disease. J. Neurol. Neurosurg. Psychiatry. 1988;51:757–66.

7. Downes JJ, Roberts AC, Sahakian BJ, Evenden JL, Morris RG, Robbins TW. Impaired extra-dimensional shift performance in medicated and unmedicated Parkinson’s disease: Evidence for a specific attentional dysfunction. Neuropsychologia. 1989;27:1329–43.

8. Wechsler D. Wechsler Memory Scale - Third Edition. Manual. San Antonio; 1997.

9. Rey A. L’Examen Clinique en Psychologie. Paris; 1964.

10. Helmstaedter C, Lendt M, Lux S. Verbaler Lern- und Merkfähigkeitstest (Verbal learning and memory test). Goettingen; 2001.

11. Bechara A, Dolan S, Hindes A. Decision-making and addiction (part II): myopia for the future or hypersensitivity to reward? Neuropsychologia. 2002;40:1690–705.

12. Dziobek I, Rogers K, Fleck S, Bahnemann M, Heekeren HR, Wolf OT, et al. Dissociation of cognitive and emotional empathy in adults with Asperger syndrome using the Multifaceted Empathy Test (MET). J. Autism Dev. Disord. 2008;38:464–73.

13. Dziobek I, Fleck S, Kalbe E, Rogers K, Hassenstab J, Brand M, et al. Introducing MASC: a movie for the assessment of social cognition. J. Autism Dev. Disord. 2006;36:623–36.

14. Linden M, Lischka A-M, Popien C, Golombek J. The Multidimensional Social Contact Circle - An interview for the assessment of the social network in clinical practice. Zeitschrift für Medizinische Psychol. 2007;16:135–43.

15. Engelmann D, Strobel M. Inequality Aversion, Efficiency, and Maximin Preferences in Simple Distribution Experiments. Am. Econ. Rev. 2004;94.

16. Charness G, Rabin M. Understanding Social Preferences with Simple Tests. Q. J. Econ. 2002;117:817–69.

17. Rösler M, Retz W, Retz-Junginger P, Thome J, Supprian T, Nissen T, et al. [Tools for the diagnosis of attention-deficit/hyperactivity disorder in adults. Self-rating behaviour questionnaire and diagnostic checklist]. Nervenarzt. 2004;75:888–95.

18. Wittchen H, Wunderlich U, Gruschwitz S, Zaudig M. Strukturiertes klinisches Interview für DSM-IV. Achse I: Psychische Störungen (SKID)(Structured Clinical Interview for DSM-IV. Axis I: Mental Disorders). Göttingen; 1997.

19. Wittchen H, Wunderlich U, Gruschwitz S, Zaudig M. Strukturiertes Klinisches Interview für DSM-IV. Achse II: Persönlichkeitsstörungen (Structured Clinical Interview for DSM-IV. Axis II: Personality Disorders). Göttingen; 1997.

20. Beck AT, Steer RA, Carbin MG. Psychometric properties of the Beck Depression Inventory: Twenty-five years of evaluation. Clin. Psychol. Rev. 1988;8:77–100.

21. Patton JH, Stanford MS, Barratt ES. Factor structure of the barratt impulsiveness scale. J. Clin. Psychol. 1995;51:768–74.

22. Cloninger CR, Przybeck TR, Svrakic DM, Wetzel RD. The Temperament and Character Inventory (TCI). A guide to its development and use. St. Louis; 1994.

23. Berth H, Cloninger CR, Przybeck TR, Svrakic DM, Wetzel RD. Das Temperament- und Charakter-Inventar (TCI). Diagnostica. 2001;47:51–3.

24. Christie R, Geis FL. Studies in Machiavellianism. New York: Academic Press; 1970.

25. Kirby KN, Petry NM. Heroin and cocaine abusers have higher discount rates for delayed rewards than alcoholics or non-drug-using controls. Addiction. 2004;99:461–71.
