# Supplementary material for: Pharmacological Cognitive Enhancement in Healthy Individuals: A Compensation for Cognitive Deficits or a Question of Personality?
Source: PLoS One. 2015 Jun 24;10(6):e0129805. doi: 10.1371/journal.pone.0129805 (PMC4479570; doi:10.1371/journal.pone.0129805)
Supplement: S1 Method — (DOCX) [file pone.0129805.s003.docx]

**S1 Method. Urine testing.**

Concentration of methylphenidate for the calibration ranged from 2 ng/mL to 1000 ng/mL and concentrations were equally distributed over the concentration range in four calibration solutions. Authentic urine samples were analyzed after protein precipitation (PP). Briefly, 300 μL of urine was mixed with 30 μL of the internal standard mixture containing methylphenidate d9 at 100 ng/mL and 30 μL of the calibration solution. Afterwards it was shaken for 5 min at 1400 rpm. Then, 900 μL of ice cold acetonitrile was added, and the mixture was shaken for 10 minutes at 1400 rpm and centrifuged for 10 min at 12000 rpm. An aliquot of 600 μL was transferred and evaporated to dryness under a gentle stream of nitrogen at 40°C. The residue was dissolved in 50 μL of a mixture of eluent A (25 mM NH4+ acetate + 0.1% acetic acid in H2O) and eluent B (0.1% acetic acid in acetonitrile). Aliquots of 10 μL of this solution were analysed by liquid chromatography-tandem mass spectrometry (LC-MS/MS). The acquisition method was Sequential Window Acquisition of all Theoretical Ion Spectra (SWATH). Quantification of ritalinic acid was done using methylphenidate calibration. For the detection of illegal drug use, the following cut-offs [1] have been applied: Cannabis, 50 ng/ml; cocaine, 150 ng/ml; and amphetamines, 500 ng/ml. Opioids, benzodiazepines, and barbiturates were not detected in our urine samples.

**Reference**

1. Substance Abuse and Mental Health Services Administration. Mandatory Guidelines for Federal Workplace Drug Testing Programs. 2008;Federal Register:71858–907.
