# Supplementary material for: Pharmacological Cognitive Enhancement in Healthy Individuals: A Compensation for Cognitive Deficits or a Question of Personality?
Source: PLoS One. 2015 Jun 24;10(6):e0129805. doi: 10.1371/journal.pone.0129805 (PMC4479570; doi:10.1371/journal.pone.0129805)
Supplement: S2 Method — (DOCX) [file pone.0129805.s004.docx]

**S2 Method. Hair testing.**

To characterize drug use over the last six months objectively, hair samples were collected and analyzed with LC-MS/MS. If participants’ hair was long enough, one sample of six cm hair (from the scalp) was taken and subsequently divided into two subsamples of three cm length. The following compounds were assessed: cocaine, benzoylecgonine, ethylcocaine, norcocaine, amphetamine, methamphetamine, MDMA, MDEA, MDA, morphine, codeine, methadone EDDP (primary methadone metabolite), tramadol, and methylphenidate. For our routine protocol for drugs of abuse analysis a three step washing procedure with water (2 minutes shaking, 15ml), acetone (2min., 10ml) and finally hexane (2min., 10ml) of hair was performed. Then the hair samples were dried at ambient temperatures, cut into small snippets and extracted in two steps, first with methanol (5ml, 16hours, ultrasonication) and a second step with 3 ml MeOH acidified with 50 µL hydrochloric acid 33 % (3 hours, ultrasonication). The extracts were dried and the residue reconstituted with 50 µL MeOH and 500 µL 0.2 mM ammonium formate (analytical grade) in water. As internal standards deuterated standards of the following compounds were used, added as mixture of the following compounds: cocaine-d3, benzoylecgonine-d3, ethylcocaine-d3, morphine-d3, MAM-d3, codeine-d3, dihydrocodeine-d3, amphetamine-d6, methamphetamine-d9, MDMA-d5. MDEA-d6, MDA-d5, methadone-d9, EDDPd3, methylphenidate-d9, tramadol-d3, oxycodone-d3, and ephedrine-d3. All deuterated standards were from ReseaChem (Burgdorf, Switzerland), the solvents for washing and extraction were of analysis grade and obtained from Merck (Darmstadt, Germany); LC-solvents were of HPLC grade and were obtained from Sigma Aldrich (Buchs, Switzerland). The LC-MS/MS apparatus was an ABSciex QTrap 3200 (Analyst software Version 1.5, Turbo V ion source operated in the ESI mode, gas 1, nitrogen (50 psi); gas 2, nitrogen (60 psi); ion spray voltage, 3500V; ion source temperature, 450°C; curtain gas, nitrogen (20 psi) collision gas, medium), with a Shimadzu Prominence LC-system (Shimadzu CBM 20 A controller, two Shimadzu LC 20 AD pumps including a degasser, a Shimadzu SIL 20 AC autosampler and a Shimadzu CTO 20 AC column oven, Shimadzu, Duisburg, Germany). Gradient elution was 3 performed on a separation column (Synergi 4µ POLAR-RP 80A, 150x2.0 with a POLAR-RP 4x2.0 Security Guard Cartridge, (Phenomenex, Aschaffenburg, Germany). The mobile phase consisted of 1mM ammonium formate buffer adjusted to pH 3,5 with formic acid (eluent A) and acetonitrile containing 1mM ammonium formate and 1 mM formic acid (eluent B). The Analysis was performed in MRM mode with two transitions per analyte and one transition for each deuterated internal standard, respectively. According to the Society of Hair Testing, the following cut-offs [1] have been applied: cocaine, 500 pg/mg; amphetamine, 200 pg/mg; and MDMA, 200 pg/mg. Opioids and other illegal drugs tested above were not detected in our hair samples.

**Reference**

1. Recommendations for hair testing in forensic cases. Forensic Sci. Int. 2004;145:83–4.
