# Supplementary material for: Pharmacological Cognitive Enhancement in Healthy Individuals: A Compensation for Cognitive Deficits or a Question of Personality?
Source: PLoS One. 2015 Jun 24;10(6):e0129805. doi: 10.1371/journal.pone.0129805 (PMC4479570; doi:10.1371/journal.pone.0129805)
Supplement: S3 Method — Significant partial correlations with a p-level below 1% are shown and marked as: **p < 0.010, ***p < 0.001. ADHD-SR: ADHD Self-Rating Scale, BIS: Barratt Impulsiveness Scale, MET: Multifaceted Empathy Test, NS: Novelty Seeking, PCE: pharmacological cognitive enhancement, RD: Reward Dependence, SCID I/II: Structural Clinical Interview for DSM-IV Axis I/II Disorders, SNQ: Social Network Questionnaire, SWM: Spatial Working Memory, TCI: Temperament and Character Inventory. (DOCX) [file pone.0129805.s005.docx]

**S3 Method. Construction of the four cognitive domain scores.**

Attention: To assess attention, we primarily focused on sustained attention by including the two RVP parameters discrimination performance A’ and total of hits [1]. In order to diversify this domain, we further added the RAVLT parameter trial 1, a supraspan measure with a strong attentional component [2].

Working memory: The SWM parameter total errors tested the capability to retain spatial information and to manipulate remembered items in the working memory [3]. The LNST score measured verbal working memory by summing up the number of correct responses [4]. The PAL first trial memory score measured visual working memory by counting the number of correctly located patterns after the first presentation [5].

Declarative memory: Three RAVLT parameters were included to assess the verbal declarative memory performance: ∑trials 1-5, delayed recall trial 7, and adjusted recognition performance p(A). Furthermore, the two PAL parameters (adjusted total of errors and adjusted total of trials) were used to capture visual declarative memory [5].

Executive functions: First, the SWM strategy score assessed the applied heuristic strategies [3] a typical feature of the executive functions. Second, the RAVLT recall consistency score is a parameter typically impaired in patients with prefrontal lesions [6,7], and related with measures of executive functions [8]. Third, the IED was used to assess visual discrimination, attentional set formation, maintenance, shifting, and flexibility [9]. The considered test parameters were the total of errors and trials adjusted to the amount of completed stages.

**References**

1. Jones GM, Sahakian BJ, Levy R, Warburton DM, Gray JA. Effects of acute subcutaneous nicotine on attention, information processing and short-term memory in Alzheimer’s disease. Psychopharmacology. 1992 p. 485–94.

2. Lezak M, Howieson D, Loring D, Hannay H, Fischer J. Neuropsychological Assessment. New York: Oxford University Press; 2004.

3. Morris RG, Downes JJ, Sahakian BJ, Evenden JL, Heald A, Robbins TW. Planning and spatial working memory in Parkinson’s disease. J. Neurol. Neurosurg. Psychiatry. 1988;51:757–66.

4. Crowe SF. Does the letter number sequencing task measure anything more than digit span? Assessment. 2000;7:113–7.

5. Sahakian BJ, Morris RG, Evenden JL, Heald A, Levy R, Philpot M, et al. A comparative study of visuospatial memory and learning in Alzheimer-type dementia and Parkinson’s disease. Brain. 1988;111:695–718.

6. Benedict RHB, Zivadinov R, Carone DA, Weinstock-Guttman B, Gaines J, Maggiore C, et al. Regional lobar atrophy predicts memory impairment in multiple sclerosis. Am. J. Neuroradiol. 2005;26:1824–31.

7. Jokeit H, Seitz RJ, Markowitsch HJ, Neumann N, Witte OW, Ebner A. Prefrontal asymmetric interictal glucose hypometabolism and cognitive impairment in patients with temporal lobe epilepsy. Brain. 1997;120:2283–94.

8. Beebe DW, Ris MD, Dietrich KN. The relationship between CVLT-C process scores and measures of executive functioning: lack of support among community-dwelling adolescents. J. Clin. Exp. Neuropsychol. 2000;22:779–92.

9. Downes JJ, Roberts AC, Sahakian BJ, Evenden JL, Morris RG, Robbins TW. Impaired extra-dimensional shift performance in medicated and unmedicated Parkinson’s disease: Evidence for a specific attentional dysfunction. Neuropsychologia. 1989;27:1329–43.
