# Supplementary material for: Pharmacological Cognitive Enhancement in Healthy Individuals: A Compensation for Cognitive Deficits or a Question of Personality?
Source: PLoS One. 2015 Jun 24;10(6):e0129805. doi: 10.1371/journal.pone.0129805 (PMC4479570; doi:10.1371/journal.pone.0129805)
Supplement: S1 Table — Data are means and standard deviations. Significant p-values are shown in bold. IED: Intra-Extra Dimensional Set-Shifting, LNST: Letter Number Sequencing Task, PAL: Paired Associates Learning, PCE: pharmacological cognitive enhancement, RAVLT: Rey Auditory Verbal Learning Test, RVP: Rapid Visual Information Processing, SWM: Spatial Working Memory. (DOCX) [file pone.0129805.s006.docx]

|  | **Controls (*n*·=·39)** | **PCE users (*n*·=·25)** | ***t*-test** | ***df*** | ***p* value** | **Cohen’s *d*** |
| --- | --- | --- | --- | --- | --- | --- |
| **Global Cognitive Index (GCI)** | 0 (0.5) | 0.1 (0.6) | 0.564 | 62 | 0.456 | 0.19 |
| **Neurocognitive domain scores** |  |  |  |  |  |  |
| Attention | 0 (0.8) | 0.2 (0.8) | 0.931 | 62 | 0.338 | 0.25 |
| Working memory | 0 (0.7) | -0.04 (0.7) | 0.039 | 62 | 0.844 | 0.05 |
| Declarative memory | 0 (0.8) | -0.01 (0.9) | 0.004 | 62 | 0.953 | 0.02 |
| Executive functions | 0 (0.7) | 0.3 (0.5) | 3.090 | 62 | 0.084 | 0.44 |
| **Neuropsychological test scores** |  |  |  |  |  |  |
| Attention |  |  |  |  |  |  |
| RVP Discrimination performance A' | 0.9 (0.04) | 0.9 (0.04) | -0.484 | 62 | 0.630 | 0.12 |
| RVP Total hits | 19.2 (4.5) | 19.7 (3.9) | -0.469 | 62 | 0.641 | 0.12 |
| RAVLT Supraspan (trial 1) | 9.9 (2.1) | 10.7 (2.5) | -1.347 | 62 | 0.183 | 0.34 |
| Working memory |  |  |  |  |  |  |
| LNST Score | 16.9 (2.7) | 16.4(3.1) | 0.631 | 62 | 0.530 | 0.16 |
| SWM Total errors | 18.0 (16.6) | 12.4 (13.1) | 1.402 | 62 | 0.166 | 0.36 |
| PAL First trial memory score | 16.7 (3.4) | 15.8 (3.7) | 1.017 | 62 | 0.313 | 0.26 |
| Declarative memory |  |  |  |  |  |  |
| RAVLT Learning performance  (∑ trials 1-5) | 65.2 (5.1) | 65.8 (7.1) | -0.371 | 62 | 0.712 | 0.10 |
| RAVLT Adjusted recognition  performance p(A) | 0.9 (0.1) | 0.9 (0.1) | 0.499 | 62 | 0.619 | 0.13 |
| RAVLT Delayed recall trial 7 | 13.8 (1.6) | 14.0 (1.4) | -0.704 | 62 | 0.484 | 0.18 |
| PAL Total errors adjusted | 7.8 (9.6) | 9.3 (9.2) | -0.641 | 62 | 0.524 | 0.16 |
| PAL Total trials adjusted | 7.9 (2.7) | 8.0 (2.7) | -0.187 | 62 | 0.852 | 0.05 |
| Executive functions |  |  |  |  |  |  |
| IED Total errors adjusted | 30.0 (38.4) | 19.4 (17.9) | 1.278 | 62 | 0.206 | 0.33 |
| IED Total trials adjusted | 104.0 (68.7) | 86.4 (32.1) | 1.196 | 62 | 0.236 | 0.31 |
| SWM Strategy score | 31.5 (5.9) | 28.1 (5.2) | 2.363 | 62 | **0**.**021** | 0.58 |
| RAVLT Recall consistency in % | 94.3 (5.0) | 94.4 (5.6) | -0.068 | 62 | 0.946 | 0.02 |

**S1 Table. Global cognitive index (GCI), the four cognitive domain z-scores, and neuropsychological test scores of stimulant-naïve healthy controls and individuals using methylphenidate for the purpose of pharmacological cognitive enhancement (PCE)**
