# Supplementary material for: Pharmacological Cognitive Enhancement in Healthy Individuals: A Compensation for Cognitive Deficits or a Question of Personality?
Source: PLoS One. 2015 Jun 24;10(6):e0129805. doi: 10.1371/journal.pone.0129805 (PMC4479570; doi:10.1371/journal.pone.0129805)
Supplement: S2 Table — Significant partial correlations with a p-level below 1% are shown and marked as: **p < 0.010, ***p < 0.001. ADHD-SR: ADHD Self-Rating Scale, BIS: Barratt Impulsiveness Scale, MET: Multifaceted Empathy Test, NS: Novelty Seeking, PCE: pharmacological cognitive enhancement, RD: Reward Dependence, SCID I/II: Structural Clinical Interview for DSM-IV Axis I/II Disorders, SNQ: Social Network Questionnaire, SWM: Spatial Working Memory, TCI: Temperament and Character Inventory. (DOCX) [file pone.0129805.s007.docx]

**S2 Table. Pearson’s product-moment correlations between test outcomes and clinical measures of social functioning with significant group differences between stimulant-naïve healthy controls (n=39) and pharmacological cognitive enhancement users (*n* = 25).**

|  | SWM strategy score,  (high performance = low score) | | | Iowa Gambling Task sum score | Iowa Gambling Task time | Machiavellianism | TCI novelty seeking (NS) sum score | TCI NS4 Disorderliness | TCI reward dependence (RD) sum score | TCI RD1 sentimentality | SCID II negativistic | SCID II antisocial | ADHD-SR sum score | BIS-11 sum score | BIS-11 attentional impulsiveness | BIS-11 attention | Dictator game, payoff B | MET cognitive empathy, correct answers | SNQ contacts |
| --- | --- | --- | --- | --- | --- | --- | --- | --- | --- | --- | --- | --- | --- | --- | --- | --- | --- | --- | --- |
| SWM strategy score,  (high performance = low score) | |  |  | |  |  |  |  |  | 0.33** |  |  |  |  |  |  |  |  |  |
| Iowa Gambling Task sum score | |  |  | |  |  |  |  |  | -0.34** |  |  |  |  |  |  |  |  |  |
| Iowa Gambling Task time | |  |  | |  | -0.49*** |  | -0.41*** |  |  | -0.33** |  |  | -0.39*** | -0.32** |  |  |  |  |
| Machiavellianism | |  |  | |  |  |  | 0.39** | -0.43*** | -0.39** | 0.46*** |  |  |  |  |  |  |  |  |
| TCI novelty seeking (NS) sum score | |  |  | |  |  |  | 0.73*** |  |  |  |  |  | 0.62*** |  |  |  |  |  |
| TCI NS4 Disorderliness | |  |  | |  |  |  |  |  |  |  | 0.46*** |  | 0.52*** |  |  |  |  |  |
| TCI reward dependence (RD) sum score | |  |  | |  |  |  |  |  | 0.70*** | -0.34** |  |  |  |  |  |  |  |  |
| TCI RD1 sentimentality | |  |  | |  |  |  |  |  |  |  |  |  |  |  |  |  |  |  |
| SCID II negativistic | |  |  | |  |  |  |  |  |  |  |  |  | 0.40*** | 0.38** |  |  |  |  |
| SCID II antisocial | |  |  | |  |  |  |  |  |  |  |  |  |  |  |  |  |  |  |
| ADHD-SR sum score | |  |  | |  |  |  |  |  |  | 0.39*** |  |  | 0.56*** | 0.73*** | 0.63*** |  |  |  |
| BIS-11 sum score | |  |  | |  |  |  |  |  |  |  |  |  |  | 0.65*** | 0.64*** |  |  |  |
| BIS-11 attentional impulsiveness | |  |  | |  |  |  |  |  |  |  |  |  |  |  | 0.90*** |  |  |  |
| BIS-11 attention | |  |  | |  |  |  |  |  |  |  |  |  |  |  |  |  |  |  |
| Dictator game, payoff B | |  |  | |  |  |  |  |  |  |  |  |  |  |  |  |  |  |  |
